# Hand-foot-genital syndrome - analysis of two cases

**DOI:** 10.5935/1518-0557.20180025

**Published:** 2018

**Authors:** Mauri J. Piazza, Almir A. Urbanetz

**Affiliations:** 1 Tocogynecology Department of the Federal University of Paraná – UFPR. Curitiba, PR, Brazil

**Keywords:** Autosomal abnormalities, Urogenital abnormalities, Hand-food-genital syndrome

## Abstract

Hand-food-genital syndrome (HFGS) is a rare genetic condition. This report describes the cases of two patients, aged 33 and 15, presenting related somatic abnormalities. HFGS stems from an autosomal anomaly linked to the HOXA 13 gene. Therapeutic procedures are discussed in order to identify the best treatment approach to the patients, as well as possible conditioning genetic anomalies.

## INTRODUCTION

Hand-foot-genital syndrome (HFGS) is an autosomal dominant hereditary disorder characterized by distinct skeletal anomalies involving the hands and the feet, associated with abnormalities of the genitourinary tract of the affected women. This anomaly was first described by Stern *et al.* (1970), when the authors examined 13 individuals from four generations in a single family. Varying degrees of duplication of the genital tract have been observed, with anatomic variations including a bicornuate uterus with a single cervix, uterus didelphys with two hemi-uteri or the presence of a septate vagina (Poznanski *et al*., 1970). The cases presented in this report describe these anomalies and showcase their wide clinical variation. Mortlock and Innis identified a HOXA 13 gene mutation as the cause for the disorder in a family diagnosed with HFGS (Mortlock & Innis, 1997). The HOXA 13 mutation has been reported in cases of HFGS and Guttmacher syndrome, two autosomal dominant congenital skeletal and urogenital syndromes (Frota Filho *et al*., 1999). Digit anomalies include short thumbs, short metacarpals, and clinodactyly. Disorders of the urinary tract include hypospadias, ectopic ureteric orifices, vesicoureteral reflux, and ureteropelvic junction obstruction.

## CASE REPORTS

### Case 1

T.A.K., 33, was referred to the Gynecology Endocrinology Service for reporting absence of menstruation. The patient weighed 66.8Kg and her height was 1.58m.

Physical examination showed normal breast development and normal axillary and pubic hair growth. Gynecological examination revealed she had normal vaginal lips and a vestibule with an intact hymen. Rectal examination found she did not have a uterus. Vaginometry was performed introducing a cotton swab in the vaginal cavity, showing a vaginal depth of about 8cm.

**Figure 1:**
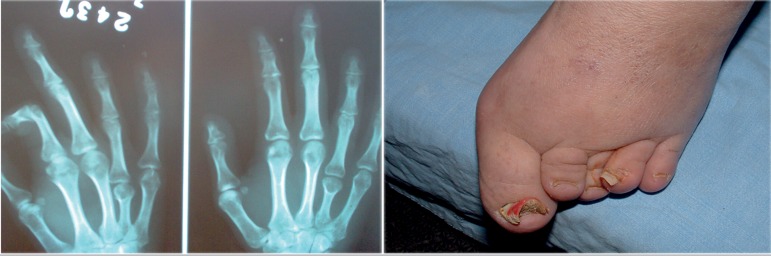
X-ray image of the hand and anomalies, patient T.A.K.: Bilateral shortening of fourth metacarpal. Several anomalies were observed in the toes

Segmental examination showed bilateral shortening of the fourth metacarpal, verified after an X-Ray of the hands and feet, a small hallux, short and pointed distal phalanges, and clinodactyly (Figure 1). Pelvic ultrasound revealed a short-sized, solid uterus of 14cm^3^ with normal shape but no uterine cavity. The right ovary measured 1.7cm and the left ovary was missing. Ultrasound examination of the urinary tract revealed a hypotrophic right kidney and a normal left kidney.

She had a normal karyotype 46, XX. FSH, LH, prolactin, and TSH plasma levels were normal.

### Case Report 2

V.R., 15, was referred to the Gynecology Endocrinology Service reporting absence of menstruation and complaining of cyclical pelvic pain. The patient weighed 46Kg and her height was 1.51m. During examination she had an episode of acute appendicitis and the ensuing surgery revealed she had a malformed uterus and blood clots in her abdominal cavity.

**Figure 2:**
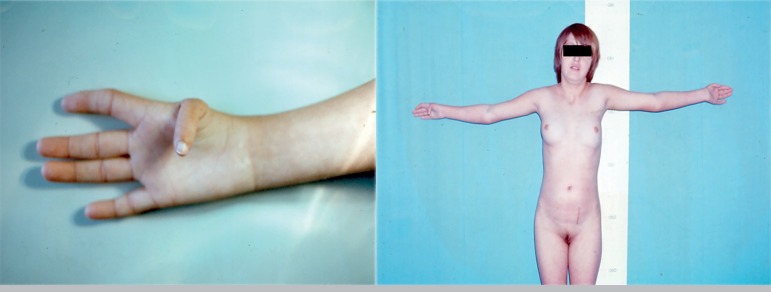
Hand anomalies and somatic aspect, patient V.R.: Agenesis of the first right metacarpal

Physical examination showed normal breast development and normal axillary and pubic hair growth (Figure 2). She had a normal vulva and an intact hymen, but an obliterated vaginal cavity prevented the insertion of a cotton swab for purposes of vaginometry. Rectal examination uncovered a medium-sized nodule in a high position deviated to the right. The patient later underwent a laparoscopy, which revealed a right-sided unicornuate uterus, left-sided hypoplastic and rudimentary horn with left fallopian tube agenesis, right-sided hematosalpinx, and normal ovaries. The left-sided abnormal uterus horn was resected. In the same procedure, the upper third of a normal vagina was visualized, although it was obliterated by a transverse septum.

Excretory urography showed only the left kidney and right renal agenesis. X-ray images demonstrated she had no sacrum or coccyx, together with malformations on both hands, agenesis of the first right metacarpal and hypoplasia of the first left metacarpal. Sex chromatin was positive.

The patient was later submitted to a McIndoe vaginoplasty for neovagina, achieving satisfactory results and restoring normal sexual function.

## DISCUSSION

A mutation causing alterations in the HOXA 13 gene function is believed to be the causal factor of hand-foot-genital syndrome (HFGS), although the exact origin of the condition is still unknown. The duplication of the genital tract observed in this syndrome might indicate direct influence of the HOXA 13 gene in the growth and/or fusion of the Müllerian ducts in human beings (Mortlock & Innis, 1997).

The HOXA 13 gene is located in the short arm of chromosome 7, between positions 15-14, also known as a transcription factor or homeobox factor, which controls the formation of several embryonic structures during embryo development. As a result, 14 mutations were previously described, 40% of which being point mutations and 60% caused by polyalanine expansions (Debeer *et al*., 2002; Innis *et al*., 2004). These mutations add extra alanines and make them abnormally long and unstable.

Duplication of the genital tract was not seen in the reported clinical cases. On the other hand, uterine hypoplasia/agenesis was observed with the aid of physical and pelvic ultrasound examination, as suggested by primary amenorrhea seen in the first case and a unicornuate uterus with an accessory left-sided rudimentary horn seen in the second case. According to the literature, the association between female genital duplication and malformations of the hands and feet is strongly correlated with HFGS and characterizes the complete spectrum of the condition (Poznanski *et al*., 1970). Skeletal abnormalities range from small feet and short hallux to hand anomalies such as shortening of the metacarpals and clinodactyly of the fifth fingers (Debeer *et al*., 2002).

Diagnostic confirmation of these cases requires chromosomal and genetic tests to identify the mutation of the HOXA13 gene (Goodman *et al*., 2000). Devriendt *et al*. (1999), Becker *et al*. (2003), Innis *et al*. (2002), Utsch *et al*. (2002), and Frisén *et al*. (2003) have described numerous cases of this condition in members of single families and isolated individuals. Imagawa *et al.* (2014) described a novel HOXA 13 mutation in a patient with severe HFGS; Wallis *et al*. (2016) described other de novo mutations in HOXA13 and NRXN1 deletion in an atypical case of HFGS with developmental delay.

Differential diagnosis includes the Holt-Oram syndrome, with common features such as hand anomalies, but different signs such as the presence of cardiac abnormalities and absence of feet and genitalia anomalies (Frota Filho *et al*., 1999; Allanson & Newbury-Ecob, 2003).

Another condition that should be included in the differential diagnosis is Guttmacher syndrome, a condition also caused by mutations of the HOXA 13 gene, in which the coexistence of genital anomalies is observed, such as hypospadias in boys (Gutmacher, 1993). However, typical alterations of this condition include hand polydactyly and no fingernail growth in the second pododactyl.

## CONCLUSION

The low prevalence of HFGS stresses the importance of adequate clinical evaluation and the addition of imaging and genetic tests in order to establish a firm diagnosis.
